# Human Rabies — South Carolina, 2011

**Published:** 2013-08-16

**Authors:** Charles E. Rupprecht, Eric Brenner, Stephanie Cox, Dana Giurgiutiu, Dan Drociuk, Jesse D. Blanton, Brett W. Petersen, Emily W. Lankau, Danielle M. Tack

**Affiliations:** Global Alliance for Rabies Control; South Carolina Dept of Health and Environmental Control; Div of High-Consequence Pathogens and Pathology, National Center for Emerging and Zoonotic Infectious Diseases; EIS officers, CDC

On December 3, 2011, a South Carolina woman visited a local emergency department (ED) with an overnight history of shortness of breath, diaphoresis, chills, and intermittent paresthesia. The patient was transferred to a referral hospital, where she became comatose and developed multiorgan failure. The patient did not report a history of an animal bite. However, family members subsequently revealed that bats had been observed in the patient’s home during the previous summer. Family members also reported that the patient had sought information on bat removal from a local county service, but was not advised of the risk for rabies associated with bat exposures and was not referred for public health consultation. CDC confirmed infection with a rabies virus variant associated with Mexican free-tailed bats (*Tadarida brasiliensis*) on December 14, after which the patient received hospice care. She died on December 19. This report summarizes the patient’s clinical course and the associated public health investigation. This case highlights the importance of strong partnerships among public health officials and diverse non–health-care partners to ensure appropriate referral of persons exposed to bats in their homes for prompt and appropriate risk assessment, postexposure prophylaxis (PEP) recommendations, and information on safe, effective, and humane bat exclusion methods.

## Case Report

On the morning of December 3, 2011, a woman aged 46 years visited a local ED with a 6-hour history of intermittent shortness of breath, diaphoresis, and chills. She also reported experiencing tingling sensations in both hands, which resolved before she sought care. Her medical history included severe heart disease with coronary artery bypass graft surgery in 2001. She reported no history of animal bite. On admission, she was alert and appropriately oriented; pulse was 94 beats per minute, blood pressure was 216/105 mmHg, and respirations were 20 breaths per minute. A complete blood count was unremarkable. Arterial blood gases showed a mild respiratory alkalosis, and serum chemistries generally were unremarkable. Imaging studies included normal computed tomography scans of the head and chest.

After 5-hours in the ED, the patient was transferred by ambulance to a large referral hospital for assessment by her cardiologist. Within 12 hours of transfer, she suffered respiratory arrest and was intubated. Her pupils became fixed and dilated during arrest. A lumbar puncture performed after resuscitation was unremarkable. The patient was transferred to the intensive-care unit. She remained intubated and sedated over the next several days and developed rhabdomyolysis, autonomic nervous system instability, and signs of multiorgan failure. Vasopressors were necessary to maintain adequate blood pressure, and hemodialysis was begun to manage acute renal failure.

Although there was no history of an animal bite, additional interviews with the family on December 8 revealed that the patient had observed bats in her home on several occasions the previous summer. Family members reported that the patient had awakened to a bat in her bedroom in August. She reportedly removed the bat by shaking it out of curtains through an open window and believed she had no direct contact with the bat. She did not seek medical attention at that time. She subsequently sought information on bat colony removal from a local county service. She was not provided with advice regarding potential rabies risks from bats occupying the home, nor was she referred to public health officials for consultation.

With this additional history, specimens were sent to CDC on December 12, 2011, for rabies virus diagnostic evaluation. Rabies virus antigens were detected in the nuchal skin biopsy by direct fluorescent antibody testing, and viral RNA was detected in both nuchal skin biopsy and saliva samples by reverse transcription–polymerase chain reaction. Sequence analysis of viral RNA was compatible with a rabies virus variant associated with Mexican free-tailed bats (*Tadarida brasiliensis*). Results were reported to the South Carolina Department of Health and Environmental Control (SCDHEC) and the referral hospital on December 14. After receiving a diagnosis of rabies, the patient received hospice care and died on December 19.

An autopsy revealed cerebral edema with uncal herniation, pulmonary edema, bronchopneumonia of the right lung, and hepatic congestion. Rabies virus antigen was detected by immunohistochemistry in multiple postmortem specimens, including brain, salivary glands, phrenic nerve, heart, liver, kidney, and adrenal gland.

## Public Health Investigation

On December 14, 2011, SCDHEC staff members met with hospital infection control, employee health, and administrative staffs to discuss rabies virus exposure risk assessments for hospital employees having contact with the patient. SCDHEC staff assessed the patient’s family, friends, coworkers, ED staff from the first hospital, and ambulance personnel who transferred the patient between hospitals. The referral hospital infection control staff performed risk assessments for their personnel. Rabies PEP was recommended to persons reporting possible transcutaneous or mucous membrane exposure to the patient’s saliva, cerebrospinal fluid, or neural tissue, based on Advisory Committee on Immunization Practices (ACIP) recommendations (1).

PEP was recommended for 22 (12%) of 188 potential contacts, including 18 health-care workers at the referral hospital and four family members. These family members had had potential exposures while caring for the patient in the hospital as well as during visits (some of them overnight) to the patient’s home during the previous months. All persons recommended to receive PEP completed the vaccine series. However, one referral hospital employee completed the series 1 week later than the schedule outlined in ACIP guidelines (1).

Veterinary public health measures also were taken for two dogs that had two possible rabies virus exposures in the patient’s home: 1) exposure to the patient’s saliva before illness onset, during the period when she might have been shedding virus, and 2) exposure to bats in the home. Both dogs had documented current rabies vaccinations. Per the Compendium of Animal Rabies Prevention and Control, both dogs were given a booster dose of canine rabies vaccine and then observed for 45 days (2). Both dogs were found to be healthy at the end of this observation period and were released from quarantine.

The patient’s home was assessed during late February 2012. Evidence of recent bat roosting was observed, including fecal material in attic and cabinet spaces adjacent to the patient’s bedroom and staining on internal and external structures near the bedroom. Openings that would allow bat ingress and egress were noted along the posterior rafters. However, no bats were observed during the inspection. The patient’s family reported that bats returned to roost in the attic during the spring of 2012. The family employed a private pest removal service to exclude the bats and seal access points. The home remained unoccupied during these remediation efforts.

### Editorial Note

This report describes the first human rabies death reported in South Carolina in more than 50 years. Human rabies has a protean clinical presentation that might be confused with other comorbidities, such as cardiac disease. Therefore, rabies should be considered for any progressive encephalitis of unknown etiology. Although human-to-human transmission has been well documented only in cases of organ or tissue transplantation, rabies virus transmission is considered possible through contamination of wounds or mucus membranes with saliva, tears, or neural tissue from infected patients (1). Use of appropriate protective equipment is vital for preventing health-care provider exposure to rabies virus when caring for patients with suspected or confirmed rabies (3). This includes use of face shields to protect mucous membranes and gloves or gowns to cover skin cuts when performing procedures, such as suctioning and spinal taps, which entail risk for exposure to infectious saliva or cerebrospinal fluid. Health-care providers should take standard precautions to prevent aerosol transmission during high-risk activities, such as intubation and suctioning (4).

What is already known on this topic?

Since 1995, over 90% of domestically acquired human rabies cases in the United States have been linked epidemiologically to bats. So-called “cryptogenic” human rabies (i.e., illness in patients who lack a definitive history of animal exposure) constitutes an increasing proportion of these bat-associated cases.

What is added by this report?

In December 2011, a woman aged 46 years was the first resident of South Carolina to die from rabies in more than 50 years. She had been hospitalized because of shortness of breath, diaphoresis, chills, and intermittent paresthesia; rabies was not suspected until family members revealed that bats had been observed in the patient’s home during the previous summer. CDC confirmed infection with a rabies virus variant associated with free-tailed bats. Of 188 family, social, and health-care contacts, 22 persons (12%) were recommended for and received postexposure prophylaxis.

What are the implications for public health practice?

Public health officials at the local, state, and national levels should work closely with non–health-care entities that receive public inquiries concerning wildlife to establish a standard referral process and regularly scheduled training about rabies risks. The diagnosis of rabies should be considered in patients hospitalized with progressive encephalopathy when other causes cannot be found or with a known history of animal exposure. This can lead to earlier adoption of staffing and infection control measures to decrease the number of health-care workers exposed to infectious body fluids or tissue for whom rabies postexposure prophylaxis might subsequently need to be provided.

Bat exposure in the home was the likely source of infection in this case. Over 90% of domestically acquired human rabies cases reported in United States since 1995 have been linked epidemiologically to bats (5). Cryptogenic human rabies (i.e., cases where a definitive history of animal exposure is lacking) constitutes an increasing proportion of these bat-associated cases (6). Rabies virus transmission can occur from seemingly minor or unrecognized bites. A complete rabies virus exposure risk assessment is recommended for any person reporting potential exposure to a bat, even in the absence of a documented bite (1).

The patient in this case sought information on bat removal but was not advised of the health risks associated with bat exposures. Lack of referral to guidance concerning health risks associated with bats living in the home was possibly a missed opportunity to prevent rabies infection. Because authority over wildlife management and animal bite reporting varies among states (7), citizens might reach out to diverse entities, including public health, animal control, law enforcement, or wildlife agencies, as initial points of contact for bat concerns. Provision of training, educational resources, and expert consultation to agencies, institutions, and organizations that provide assistance with wildlife concerns is a valuable public health service. Such service requires strong partnerships and clear communication among public health officials and diverse community partners.

Human rabies is preventable by avoiding contact with animal vectors and by receiving prompt and appropriate wound care and PEP after a suspected rabies virus exposure. Public health officials should work closely with non–health-care partners that receive public inquiries concerning wildlife to establish a standard referral process and regularly scheduled training about rabies risks. The public should also be educated about the risk for rabies from bat exposures, and options for the safe removal and exclusion of bats from human dwellings.
